# Prenatal Exposure to Organophosphorus Pesticides and Preschool ADHD in the Norwegian Mother, Father and Child Cohort Study

**DOI:** 10.3390/ijerph19138148

**Published:** 2022-07-02

**Authors:** Cherrel K. Manley, Gro D. Villanger, Cathrine Thomsen, Enrique Cequier, Amrit K. Sakhi, Ted Reichborn-Kjennerud, Amy H. Herring, Kristin R. Øvergaard, Pal Zeiner, Kyle R. Roell, Lawrence S. Engel, Elizabeth M. Kamai, Jake Thistle, Amber Hall, Heidi Aase, Stephanie M. Engel

**Affiliations:** 1 Department of Epidemiology, Gillings School of Global Public Health, University of North Carolina at Chapel Hill, Chapel Hill, NC 27599, USA; ccherrel@live.unc.edu (C.K.M.); kyle.roell@unc.edu (K.R.R.); larry.engel@unc.edu (L.S.E.); jthistle@live.unc.edu (J.T.); ambermh@live.unc.edu (A.H.); 2 Department of Child Health and Development, Division of Mental and Physical Health, Norwegian Institute of Public Health, 0456 Oslo, Norway; gro.dehli.villanger@fhi.no (G.D.V.); heidi.aase@fhi.no (H.A.); 3 Department of Food Safety, Division of Climate and Environmental Health, Norwegian Institute of Public Health, 0456 Oslo, Norway; cathrine.thomsen@fhi.no (C.T.); ecequier@hotmail.com (E.C.); amritkaur.sakhi@fhi.no (A.K.S.); 4 Department of Mental Disorders, Division of Mental and Physical Health, Norwegian Institute of Public Health, 0456 Oslo, Norway; ted.reichborn-kjennerud@fhi.no; 5 Department of Statistical Science and Global Health Institute, Duke University, Durham, NC 27708, USA; amy.herring@duke.edu; 6 Division of Mental Health and Addiction, Oslo University Hospital, 0424 Oslo, Norway; kristin.r.overgaard@gmail.com (K.R.Ø.); sbzeip@ous-hf.no (P.Z.); 7 Institute of Clinical Medicine, University of Oslo, 0315 Oslo, Norway; 8 Department of Population and Public Health Sciences, Keck School of Medicine, University of Southern California, Los Angeles, CA 90007, USA; kamai@usc.edu

**Keywords:** organophosphorus pesticide, preschool attention-deficit hyperactivity disorder, prenatal exposure, the Norwegian Mother, Father and Child Cohort Study, medical birth registry of Norway

## Abstract

Prenatal organophosphorus pesticide (OPP) exposure has been associated with child attention-deficit/hyperactivity disorder (ADHD) in agricultural communities and those that are exposed to residentially applied insecticides. To examine this association in populations that are exposed primarily through diet, we estimate the associations between prenatal OPP exposure and preschool ADHD in the Norwegian Mother, Father and Child Cohort Study (MoBa), and describe modification by paraoxonase 1 (*PON1*) gene variants. We used participants from the MoBa Preschool ADHD Sub-study (*n* = 259 cases) and a random sample of MoBa sub-cohort participants (*n* = 547) with birth years from 2004 to 2008. Prenatal urinary dialkylphosphate (DAP) metabolites (total diethylphosphate [∑DEP] and total dimethylphosphate [∑DMP]) were measured by an ultra-performance liquid chromatography-time-of-flight system and summed by molar concentration. Maternal DNA was genotyped for coding variants of *PON1* (Q192R and L55M). We used a multivariable logistic regression to calculate the odds ratios (OR) and 95% confidence intervals, adjusted for maternal education, parity, income dependency, age, marital status, ADHD-like symptoms, pesticide use, produce consumption, and season. We found no associations between DAP metabolite concentrations and preschool ADHD. The adjusted ORs for exposure quartiles 2–4 relative to 1 were slightly inverse. No monotonic trends were observed, and the estimates lacked precision, likely due to the small sample size and variation in the population. We found no evidence of modification by *PON1* SNP variation or child sex. Maternal urinary DAP concentrations were not associated with preschool ADHD.

## 1. Background

Organophosphorus pesticides (OPPs) are widely used in commercial agriculture for pest control. They function through irreversibly inhibiting the acetylcholinesterase enzyme, disrupting neurotransmission [1]. At high doses, OPP exposure in humans can result in organophosphate-induced delayed polyneuropathy and acute cholinergic toxicity [2]. Although the biological mechanisms are not fully understood, a number of prospective, longitudinal cohorts of perinatal OPP exposure have also demonstrated that low dose exposure may cause adverse neurodevelopmental outcomes among children [3,4,5,6].

Exposure in the general population is thought to occur to a large extent through diet, primarily due to the consumption of contaminated produce. Several studies have reported associations between measured urinary OPP metabolites and fruit and vegetable consumption [7,8,9,10,11], and that the consumption of organic produce significantly reduces urinary concentrations of OPP metabolites [7,11,12,13,14,15]. Due to the low pest population and limited agriculture in Norway, the application of pesticides is minimal; however, prior research suggests that the Norwegian population may be exposed to pesticides via consumption of imported produce [16]. In fact, despite the limited within-country use of pesticides, urinary OPP metabolite levels among Norwegians are similar to residents of countries with higher levels of agricultural activity [17,18].

Prior studies have found that prenatal OPP exposure results in cognitive and behavioral deficits in highly exposed agricultural communities, as well as in populations that are exposed to residential applications prior to the de-registration of chlorpyrifos and diazinon by the United States Environmental Protection Agency (US EPA) [3,4,6,17,19,20,21,22,23]. With respect to attention-deficit/hyperactivity disorder (ADHD) symptoms (hyperactive-impulsivity and/or inattention) specifically, prenatal OPP exposure has been linked to increases in attention problems and ADHD index scores, as well as poorer working memory, processing speed, and intelligence quotient (IQ) scores in offspring [4,6,19,22,23,24]. However, few studies have been conducted in populations experiencing mainly dietary exposure, which is more reflective of typical exposure patterns in the general population. Additionally, studies that have assessed attention problems or psychometric measures of executive function in the preschool and early childhood periods have relied heavily on parentally reported inventories of child behavior or performance-based assessments, without a clinical assessment of ADHD [6,19,22,23]. To address these limitations of the existing literature, we sought to study the relationship between prenatal OPP exposure and clinically evaluated preschool ADHD in a population primarily experiencing indirect OPP exposure through dietary sources.

Additionally, no prior study addressing ADHD symptoms has considered the potentially modifying influence of functional variants in the paraoxonase 1 (*PON1*) gene, which has previously been shown to modify associations of OPPs with other neurodevelopmental endpoints [25,26,27]. PON1 is a major enzyme that is responsible for the metabolism of organophosphorus compounds. There are several well-characterized single-nucleotide polymorphisms (SNPs) in the *PON1* gene that affect the speed of metabolism and excretion [28,29,30,31]. The Q192R polymorphism results in OPP substrate-specific differences in PON1 catalytic speeds, while the L55M polymorphism is reported to be associated with variation in the plasma PON1 protein levels [28]. Given prior evidence of important gene–environment interactions, in addition to the sparse literature on modification of the association between OPPs and ADHD symptoms, we sought to assess the influence of SNPs in *PON1* in the current investigation. Additionally, modification by child sex at birth was also of interest due to prior reports of differential effects of prenatal OPP exposure on child neurodevelopment and variation in prevalence of ADHD diagnoses by sex [6,32].

In this study, we leverage a high-quality investigation of preschool ADHD in the Norwegian Mother, Father, and Child Cohort (MoBa) to examine associations between prenatal exposure to OPPs and clinically evaluated preschool ADHD. Additionally, we aim to assess modification by genetic variation in *PON1*, as well as by child sex. We hypothesize that prenatal OPP exposure increases the risk of preschool ADHD, and that slow metabolizers of OPPs experience higher risks of ADHD with OPP exposure as compared to faster OPP metabolizers.

## 2. Methods

### 2.1. Study Population

The MoBa study is an ongoing prospective, population-based cohort of Norwegian-speaking women, conducted by the Norwegian Institute of Public Health (41% participation rate) [33]. In MoBa, women were enrolled between 1999 and 2008 at approximately 17 weeks gestation, at which time their maternal urine samples were also collected. Mothers completed questionnaires at 17, 22 (diet only), and 30 weeks gestation. Birth date and outcome information was collected through linkage with the Medical Birth Registry of Norway, a validated national health registry containing information about all births in Norway since 1967 [34]. Written informed consent was obtained from each MoBa participant at the time of recruitment. The establishment of MoBa and the initial data collection was based on a license from the Norwegian Data Protection Agency with approval from The Regional Committees for Medical and Health Research Ethics. The MoBa cohort is currently regulated by the Norwegian Health Registry Act. The current study was approved by The Regional Committees for Medical and Health Research Ethics (2012/985) and the University of North Carolina Office of Human Research Ethics (12-0703).

### 2.2. MoBa Preschool ADHD Sub-Study

The Preschool ADHD sub-study is a sub-study within MoBa consisting of births that took place on 1 April 2004 through 31 December 2008 of mothers who completed the 3-year questionnaire and lived in close proximity to Oslo. Exclusions for this study included diagnosis with Cerebral Palsy or Down’s syndrome, or symptoms of autism spectrum disorder that were substantial enough to qualify the child for the concurrent MoBa Autism Birth Cohort study [35].

When the child was 3 years old, the mothers completed a MoBa questionnaire that included 11 items about ADHD, including six items from the Child Behavior Checklist/1.5–5 and five items from the DSM-IV-TR criteria for ADHD [36,37,38]. Numeric scores were assigned to the responses for each item and summed to create a quantitative index of responses for this subset of questions [39]. Children with scores at or above the 90th percentile on these 11 items (*n* = 2798), along with children randomly selected from those that were eligible for the Preschool ADHD Sub-study (*n* = 654), were invited to participate in clinical assessments. In total, about 35% agreed to participate in a 1-day clinical assessment that included a diagnostic interview for preschool ADHD (further described below). The clinical assessments were completed between 2007 and 2011 (child mean age: 3.5 years; range: 3.1–3.8 years). Of those who participated, 830 had available maternal prenatal urine samples stored in the MoBa biobank and were therefore eligible to participate in this current study.

### 2.3. Preschool ADHD Diagnostic Interview

Clinical assessments were completed using the Preschool Age Psychiatric Assessment [40]. Only symptoms lasting three or more months were counted as present. Trained graduate psychology students conducted the interviews under the supervision of child psychologists or psychiatrists. As an inter-rater reliability check, separate raters, blinded to other instruments that were included in the clinical assessment, rescored audiotapes of 79 randomly selected interviews [41]. The average intra-class correlation was 0.98 for the total number of ADHD symptoms. We defined ADHD as present when the children fulfilled or nearly fulfilled the DSM-IV-TR-symptom criteria for the predominantly hyperactive/impulsive, predominantly inattentive, or combined presentations reported by parents to be pervasive across at least two settings. From this assessment, 260 children were found to fulfill or nearly fulfill ADHD criteria, hereafter referred to as Preschool ADHD cases.

### 2.4. MoBa Reference Population

Among the population eligible to participate in the Preschool ADHD Sub-study who also had prenatal maternal urine and blood available (*n* = 16,577), we randomly sampled a reference population (i.e., sub-cohort), which was frequency matched to the preschool ADHD cases based on year of birth (*n* = 556). Seven participants who were randomly sampled into the reference population were also Preschool ADHD cases. Given the small number of overlapping observations, we treated them only as cases in the current analysis, and therefore our reference population consists of 549 participants.

### 2.5. Measurement of Dialkylphosphate (DAP) Metabolites

To estimate prenatal organophosphorus-pesticide exposure, we measured six dialkylphosphate (DAP) metabolites in spot urines that were collected at approximately 17 weeks gestation: diethylphosphate (DEP); diethylthiophosphate (DETP); diethyldithiophosphate (DEDTP); dimethylphosphate (DMP); dimethylthiophosphate (DMTP); and dimethyldithiophosphate (DMDTP). Case and sub-cohort samples were randomly allocated across analytic batches. The DAP metabolites were measured using an ultra-performance liquid chromatography-time-of-flight system (UPLC-TOF) [18]. In-house control urine samples, at two concentrations, were included for internal quality control (QC), as well as 4-6 laboratory-blinded pooled QCs per analytic batch. Values below the limit of detection (LOQ) were imputed from a log-normal distribution that was truncated at the LOQ. To calculate the total diethyl- (∑DEP) and total dimethylphosphate (∑DMP) metabolite concentrations, we summed the individual metabolites within groups by molar concentration. We excluded DEDTP from ∑DEP because 99% of the values were less than the LOQ. Specific gravity was measured using a pocket refractometer (PAL-10S) from Atago to account for urinary dilution. We standardized the ∑DEP and ∑DMP concentrations for specific gravity, as previously described [42]. Two participants from the sub-cohort and one from the case group were missing DAP measurements and, thus, were excluded from all analyses. Assessment of the QC samples and imputation below the LOQs were conducted using R version 3.5.1.

### 2.6. Genotyping of Paraoxonase 1 L55M and Q192R

We collected maternal blood samples in EDTA tubes at participants’ 17th week of gestation and separated the plasma, before shipping overnight to the MoBa Biobank [43]. The samples were stored at −80 °C prior to analysis [44]. Maternal DNA was extracted using the FlexiGene kit (Qiagen, Hilden, Germany) and stored at −20 °C. Genotyping was conducted at the Norwegian University of Life Sciences (NMBU) using the Sequenom MassARRAY IPLEX^®^ platform (iPlex, Adelaide, SA, Australia) [45].

### 2.7. Covariates

Covariate data were obtained through maternal self-report in MoBa questionnaires and from the Medical Birth Registry of Norway. The birth registry was used to obtain maternal age at delivery, in years, which was treated as a continuous measure. Birth year and child sex (male/female) were also obtained through this registry. Maternal education (less than 4-year university or technical degree completed; 4-year university or technical degree completed; more than 4-year university or technical degree completed; and any other education level); marital status during pregnancy (married, divorced/separated, cohabitant, widow, single, other); parity (primiparous versus multiparous); maternal income dependency (3-levels: total income < 400,000 NOK and dependent on maternal income; total income ≥ 400,000 NOK and dependent on maternal income; not dependent on maternal income); any self-reported contact with weed killers, insecticides, or fungicides either at work or in leisure time within six months prior to the 17th week of gestation (yes/no); and any maternal smoking and alcohol consumption during pregnancy up until the 17th week of gestation (yes/no) were all obtained using MoBa questionnaire 1, which was completed at approximately the 17th week of pregnancy. Total fruit consumption and raw vegetable consumption were measured using a food frequency questionnaire that was completed by MoBa women between the 17th and 22nd week of pregnancy, where mothers reported the number of fresh fruits they had eaten on average and how often they had eaten raw vegetables on average per day, week, and month since becoming pregnant. From this information, we created summary variables for servings of raw vegetables and of fresh fruit per day. We obtained maternal ADHD-like symptoms using the ADHD self-report scale, which was included in the 3-year questionnaire, and used a score of ≥4 as the threshold for ADHD-like symptoms (score of 4 or more vs. less than 4) [46]. The season of urine collection was determined by the month of urine donation, and defined as summer (June–August); fall (September–November); winter (December–February); or spring (March–May).

### 2.8. Statistical Analysis

We assessed the distributions of covariates individually and according to case/subcohort status. We used multivariable logistic regression to obtain the odds ratios (ORs) and 95% confidence intervals (95% CI) of the association between measured DAP metabolite concentrations and preschool ADHD, adjusted for confounding variables that were determined by a directed acyclic graph (DAG) [47]. Based on our DAG, potential confounders were maternal education, parity, maternal income dependency, maternal age, marital status, maternal ADHD-like symptoms, occupational/recreational pesticide use, fresh fruit and vegetable consumption, and season of urine collection. We did not adjust for marital status because approximately 99% of the participants were either married or cohabitant during their pregnancy.

Our final model (model 1), consisting of all potential confounders, adjusted for maternal education, parity, maternal income dependency, maternal age, maternal ADHD-like symptoms, occupational/residential pesticide use, fruit and vegetable consumption, and season. We additionally compared models across three minimally sufficient adjustment sets: (2) maternal age, maternal education, and parity; (3) maternal income dependency, maternal education, and parity; (4) maternal age, maternal education, maternal ADHD-like symptoms, and parity. Lastly, we examined a 5th model, consisting of all potential confounders, excluding those that were eliminated using change in estimate selection (10% change in the effect size) [48]. This model adjusted for maternal education, parity, maternal income dependency, maternal age, maternal ADHD-like symptoms, and fruit and vegetable consumption.

The DAPs were modeled both continuously and in quartiles to assess functional form. Our primary tables present results from models including all the potential confounders that were identified on the DAG. However, parsimonious models with more limited adjustment sets are included in Supplementary Material Table S2. All models were co-adjusted for ∑DEP and ∑DMP concentrations and were analyzed in complete case form (i.e., individuals with missing covariate data were excluded). Model 1 excluded 11% of participants due to missing covariate data, while models 2, 3, 4, and 5 excluded 1%, 4%, 3%, and 7% of participants, respectively.

To assess the effect measure modification by the *PON1* genotype, we included interaction terms for SNP genotype*DAP metabolite (∑DEP and ∑DMP) concentrations in our final model. Separately, to assess modification by child sex on the multiplicative scale, we included interaction terms for child sex*DAP metabolite (∑DEP and ∑DMP) concentrations in our final model. We considered the effect measure modification to be statistically significant at a *p*-value of <0.05, as determined a priori. The current analysis is based on version 9 of the MoBa quality-assured data files. We conducted all statistical analyses using SAS 9.4 software (Cary, NC, USA).

## 3. Results

Characteristics of the preschool ADHD case and sub-cohort populations are included in Table 1 (demographics) and Table 2 (behavioral characteristics). The mean maternal age at birth in both groups was approximately 30 years. Self-reported elevated maternal ADHD symptoms were higher among preschool ADHD cases relative to the sub-cohort (12.5% versus 3.9%). The mothers of ADHD cases were more likely to report both a family dependence on maternal income and a lower maternal income. ADHD cases were more often first-born (59.9% versus 49.2%), which may be due in part to the study requirement that cases participate in an in-person clinical assessment. The mothers of ADHD cases were more often smokers, compared to the mothers of sub-cohort children (26.3% versus 13.9%), while the frequency of maternal alcohol use during pregnancy was similar across the groups. The prenatal consumption of fruits and vegetables (servings per day) was similar across the groups. Overall, the number of women reporting exposure to pesticides at home, at work, or in their leisure activities was low, but slightly higher for cases compared to sub-cohort children (6.1% versus 4.8%). Children in the ADHD case group were more likely to be male (56% versus 50.1%). Finally, the seasonal distribution of urine collection was similar for both groups. Less than 5% of values were missing for all covariates, except for parity, maternal alcohol use, and pesticide exposure (all <10% missing).

The raw DAP metabolite concentrations and the distributions of the measured maternal urinary molar DAP sums accounting for specific gravity are presented in Table 3. Concentrations of DEP and DMTP were detectable in the majority of the study population (Table 3), while DETP and DMP were detectable in about half of the study population. Although rare, DMDTP was detected in about 15% of subjects and therefore was included in ∑DMP. DEDTP was rarely detected (<1% of participants) and therefore was not included in ∑DEP. For each metabolite that was measured, and ∑DMP and ∑DEP, the geometric mean concentrations were higher in the sub-cohort as compared to the case group (Supplementary Material Table S1). Median and maximum exposures to ∑DMP metabolites were higher in the study population compared to ∑DEP metabolite exposures (Table 3).

We observed no association between maternal urinary ∑DEP or ∑DMP concentration and child preschool ADHD in multivariable models that were adjusted for maternal education, parity, maternal income dependency, maternal age, maternal ADHD-like symptoms, occupational/residential pesticide use, fruit and vegetable consumption, and season (Table 4). In general, the adjusted odds ratios for quartiles (Q) 2–4 relative to Q1 were slightly inverse, however there were no monotonic trends and estimates hovered around the null. In sensitivity analyses where models consisted of reduced adjustment sets, associations were consistent with an overall null association (Supplementary Material Table S2). We also observed no modification by variation in the *PON1* SNP genotype or child’s sex at birth on the multiplicative scales (Table 5).

## 4. Discussion

In this nested study of MoBa, we found no increased odds of preschool ADHD in relation to prenatal urinary OPP metabolite concentrations. Furthermore, we did not find evidence that the associations were modified by the *PON1* genotype, or that they varied by child sex.

The literature linking prenatal OPP exposure with ADHD is mixed [6,19,20,21,24]. Several analyses have been conducted within the Center for the Health Assessment of Mothers and Children of Salinas (CHAMACOS) cohort, a birth cohort in an agricultural region of California. Eskenazi et al. (2007) reported no associations between prenatal urinary DAP concentrations and Child Behavior Checklist (CBCL)-based measures of attention problems or ADHD at 2 years of age [24]; however, Marks et al. (2010) found prenatal urinary DAPs to be positively associated (i.e., more inattention symptoms with increased exposure) with these outcomes at 5 years (and 3.5 years for boys only) in the same community [6]. The latter study additionally reported positive associations between prenatal urinary DAPs and attention problems that were ascertained through computerized testing (Conners’ Kiddie Continuous Performance Test (K-CPT)) and a psychometrician report (via the Hillside Behavior Rating Scale) at 5 years, as well as evidence of modification by sex, with boys experiencing stronger effects [6]. In this same cohort, researchers have reported associations between prenatal OPP exposure and characteristics of ADHD, including lower working memory, processing speed, and full-scale intelligence quotient (FSIQ) scores at 7 years, based on assessment using the Wechsler Intelligence Scale for Children (WISC-IV) [4,19]. Researchers have also identified adverse associations between prenatal residential proximity to OPP application sites and FSIQ at 7 years, working memory at 10 years, and brain activation during tasks of executive function at 15–17 years [4,20,21]. Although these findings provide mixed evidence of associations between prenatal OPP exposure and cognitive mechanisms that may be related to ADHD, CHAMACOS participants were living in farmworker communities and were more likely to have experienced substantial direct exposure to pesticides. Therefore, these results may have limited generalizability to general population exposures, which tend to be lower than in agricultural communities. In the case of our study, we find that although the average ∑DEP levels were similar to those that were reported in CHAMACOS, the average ∑DMP levels in our study were slightly lower. It is also important to note that the modes and types of OPP exposure, as well as the background regulatory environments likely vary across populations.

Other studies in inner city cohorts that were enrolled prior to the withdrawal of chlorpyrifos and diazinon for residential applications in the United States have found deficits in working memory in relation to DEP exposure; similar associations were found in relation to umbilical cord blood measures of chlorpyrifos [22,23,49]. Studies have also examined relationships between OPP exposure and behavior or social cognition using parent-reported inventories. In a study of children born in New York City (1998–2002), Rauh et al. (2006) found that children whose mothers had higher chlorpyrifos concentrations during pregnancy were significantly more likely to have attention problems and ADHD problems based on the CBCL scale at age 3 [23]. In a separate study of New York City residents, prenatal DAP metabolite concentrations were associated with more adverse scores on the Social Responsiveness Scale (SRS) at age 7–9 years, particularly among boys [50]. This finding is relevant since impaired social functioning is also common among children with ADHD [51]. However, the exposure patterns of these cohorts, including a high likelihood of direct exposure to parent compounds from residential OP insecticide use, may not be directly comparable to that of MoBa, in which exposures are likely to occur primarily via the diet. Similar to our study, Millenson et al. (2017) reported no association between maternal urinary DAP metabolites and SRS scores in the Health Outcomes and Measures of the Environment (HOME) Study, a birth cohort in Cincinnati, Ohio that was enrolled after the ban on residential applications, where, like our study, OPP exposures were more likely to occur through diet [52]. We find that average ∑DEP levels estimated in our study are similar to those that were reported in the aforementioned US, city-based cohorts, while the average ∑DMP levels in our study are slightly higher.

A recent study that was conducted in the Generation R cohort in the Netherlands reported similar results to ours. Specifically, DAP metabolite concentrations were not associated with ADHD traits measured using CBCL scales at ages 6, 8, and 10 years (born 2002–2006) [53]. In the Generation R cohort, exposure to organophosphorus pesticides was measured at three times during pregnancy, which addressed the rapid metabolism of these compounds. Interestingly, DAP metabolite concentrations in the Generation R study are much higher (10 nmol/L higher for median ∑DEP and twice the magnitude of median ∑DMP estimates in the present study) than those in our study. Van den Dries et al. (2019) hypothesize that the high exposure levels may be related to higher consumption of produce in this population or to farming practices in the Netherlands, where more pesticides are used per square kilometer of farmland than in most other countries [10]. Despite this high exposure, they found no overall association of DAP concentrations with ADHD traits. However, the Generation R cohort was not able to account for confounding by the presence of maternal symptoms of ADHD. This is important because ADHD has a strong genetic component, which may confound estimates of prenatal exposure through differences in behaviors or diet [54,55,56]. In addition, the Generation R cohort did not utilize clinically assessed ADHD but relied on maternal report using the CBCL.

Although we found no evidence of effect measure modification by the *PON1* genotype, prior studies have reported significant heterogeneity in associations of prenatal DAPs with psychomotor and mental development indices (PDI/MDI) by the *PON1* genotype, and found stronger inverse associations among individuals who were slow metabolizers of OPPs [3,17]. However, other studies have not found substantial effect measure modification by the PON1 genotype [52,57,58]. Similar to our study, prior studies found no modification of associations of prenatal urinary DAPs with SRS scores and nonverbal IQ, MDI, PDI, and pervasive developmental disorder (PDD) scale scores by genetic variation in PON1 [52,57,58]. Although differences in PON1 genotypes are associated with differences in enzymatic activity, substantial variability in enzymatic activity has been demonstrated within genotypes [59]. For this reason, it may be more appropriate to assess the PON1 phenotype as a modifier of relationships between prenatal OPP exposure and child neurodevelopmental outcomes.

Our study is distinct from the previously described studies because we assessed preschool ADHD using a validated diagnostic interview for preschool-aged children, and considered plausible modifiers (sex and *PON1* genotype) of the association between prenatal OPP exposure and child neurodevelopment. Our study leveraged a high quality standardized clinical assessment with ADHD ascertainment that was based on a standardized diagnostic interview; well-measured confounders, including information on fruit and vegetable consumption during pregnancy and occupational and recreational use of pesticides; and adjustment for maternal symptoms of ADHD. Another major strength was our consideration of genetic determinants of effect measure modification by including mothers’ *PON1* SNPs L55M and Q192R variants, since both are known to affect the speeds of metabolism and excretion, and Q192R has been shown to modify neurodevelopmental associations with DAPs in prior studies [3,17]. In addition, our study design, which included a birth-year stratified random sample of all the children that were eligible for the preschool ADHD sub-study, allowed us to estimate measures of association while accounting for maternal factors that may have been predictive of participation in the clinical assessment, for example parity, maternal education, marital status, and maternal income dependency. Finally, the route of exposure that is represented in this study, specifically exposure occurring predominantly through conventionally grown fruits and vegetables, likely reflects a typical route in a general non-farming population.

While our study leveraged a high quality standardized assessment of ADHD, ADHD that is diagnosed in the preschool period may have only moderate diagnostic stability [60], and the trajectories and predictive power of ADHD symptoms in the preschool period have been shown to vary across ages [61,62]. Therefore, outcome misclassification of an underlying ADHD phenotype is a possibility when examining preschool-age children. We were limited to one maternal urine donation at 17 weeks gestation, and OPPs are rapidly metabolized and eliminated; therefore, a single measurement of exposure may inadequately reflect exposure over the entire window of pregnancy [63]. While misclassification in exposure is a possibility, our findings are in line with the results of Van den Dries et al. (2019), who collected three urine samples during pregnancy [53]. Urinary DAP concentrations may also be imperfect biomarkers of parent OPP exposure, particularly for dietary exposure sources. Prior studies have reported higher concentrations of preformed DAPs compared to the toxic parent compounds on produce, and preformed DAPs are not neurotoxic; therefore, dietary exposure includes a mix of parent compound and preformed DAP metabolites, which are indistinguishable by their urinary biomarkers [64,65,66]. Lastly, DAP metabolites are nonspecific, so we are limited in our inference about the impact of specific parent OP pesticides, which vary in toxicity. For this reason, comparing associations across populations with only urinary DAP measures is challenging, as the underlying contribution of specific parent compounds may vary, and parent compounds vary in their toxicity.

Given their short growing season, most of the produce in Norway is imported from other countries, with Spain, the Netherlands, Italy, South Africa and Peru as key import partners [67]. Therefore, growing practices in these countries are a determinant of exposure in the general Norwegian population. However, there are few attractive alternatives to urinary DAP metabolites as a marker of OPP exposure. Pesticide-specific biomarkers tend to have low detection frequencies, making them infeasible to implement in population-based studies with dietary exposure. DAP metabolites, though non-specific, integrate exposure to all parent compounds—therefore, their high exposure detection frequencies make them practical choices for general population exposures. Furthermore, we must consider that due to the birth year of the participants in our study (2004–2008), regulations and OPP exposure patterns may differ for pregnant people and their children in later years. Lastly, we may not have had the sufficient statistical power to identify significant effect measure modification, especially considering the possible exposure and outcome misclassification.

In conclusion, prenatal urinary DAP metabolites were not associated with an increased risk of preschool ADHD in this nested case–cohort study of the MoBa cohort. A key determinant of pesticide exposure in the Norwegian population is consumption of fresh fruit and vegetables [9]. Thus, our research does not support a strong impact of prenatal dietary exposure to OPPs on preschool ADHD risk in a general, non-agricultural population.

## Figures and Tables

**Table 1:** Demographic characteristics of study population in nested case–cohort study of preschool attention-deficit hyperactivity disorder in the Norwegian Mother, Father and Child Cohort (MoBa), birth years 2004–2008.

	Preschool ADHD Cases (*n* = 259)		Sub-Cohort (*n* = 547)	
	*n*	%	*n*	%
	(Mean, SD)		(Mean, SD)	
Maternal age at delivery (years)	(30.0, 4.05)		(30.9, 4.23)	
<26	32	12.4	46	8.4
26–30	110	42.5	205	37.6
31–35	99	38.2	218	40.0
>35	18	7.0	76	13.9
Missing (*n*)	0		2	
Maternal education				
<4-year university	91	35.3	121	22.4
4-year university	108	41.9	236	43.7
>4-year university	59	22.9	183	33.9
Missing (*n*)	1		7	
Maternal ADHD-like symptoms *				
No	225	87.6	514	96.1
Yes	32	12.5	21	3.9
Missing(*n*)	2		12	
Marital status during pregnancy				
Married	128	49.6	298	55.0
Cohabitant	130	50.4	244	45.0
Missing (*n*)	1		5	
Maternal income dependence				
Family not dependent on maternal income	34	13.6	105	19.8
Dependent on maternal income ≥400,000 NOK	88	35.2	221	41.6
Dependent on maternal income <400,000 NOK	128	51.2	205	38.6
Missing (*n*)	9		16	
Parity				
Primiparous	155	59.9	268	49.2
Multiparous	104	40.2	277	50.8
Missing (*n*)	20		44	
Child sex				
Male	145	56.0	274	50.1
Female	114	44.0	273	49.9
Missing (*n*)	0		0	

Note: ADHD, attention-deficit hyperactivity disorder; SD, standard deviation; NOK, Norwegian Kroner; * Maternal ADHD self-report scale with cut-off at 4 (yes—score of 4 or more, no—score of less than 4).

**Table 2:** Behavioral characteristics of study population in nested case–cohort study of preschool attention-deficit hyperactivity disorder in the Norwegian Mother, Father and Child Cohort (MoBa), birth years 2004–2008.

	Preschool ADHD Cases (*n* = 259)		Sub-Cohort (*n* = 547)	
	*n*	%	*n*	%
	(Mean, SD)		(Mean, SD)	
Any maternal smoking during pregnancy up until the ~17th week of gestation				
Yes	61	23.6	75	13.9
No	197	76.4	466	86.1
Missing (*n*)	1		6	
Any maternal alcohol use during pregnancy up until the ~17th week of gestation				
Yes	32	13.4	65	12.9
No	207	86.6	438	87.1
Missing (*n*)	20		44	
Fresh fruit consumption during pregnancy up until ~22 weeks gestation (servings per day)	(2.0, 1.4)		(2.1, 1.3)	
Missing (*n*)	4		7	
Raw vegetable consumption during pregnancy up until ~22 weeks gestation (servings per day)	(1.4, 0.8)		(1.4, 0.9)	
Missing (*n*)	8		21	
Self-reported pesticide exposure *				
Yes	15	6.1	25	4.8
No	230	93.9	495	95.2
Missing(*n*)	14		27	
Season of prenatal urine collection				
Winter	70	27.3	156	28.5
Fall	56	21.6	119	21.8
Spring	66	25.5	135	24.7
Summer	67	25.9	137	25.1
Missing (*n*)	0		0	

Note: ADHD, attention-deficit hyperactivity disorder; SD, standard deviation, * Maternal self-report of contact with weed killers, insecticides, or fungicides either at work or in leisure time during the six months prior to 17th week of gestation.

**Table 3:** Prenatal urinary organophosphorus-pesticide metabolite concentration distribution in a nested case–cohort study of preschool attention-deficit hyperactivity disorder in the Norwegian Mother and Child Cohort (MoBa), birth years 2004–2008.

OP metabolite	Population	N	LOQ	%>LOQ	Min	25%	50%	75%	Max
DEP (ng/mL)	Case	259	1.089	67.3	<LOQ	<LOQ	1.67	3.27	32.8
	Sub-cohort	548	1.089	67.4	<LOQ	<LOQ	1.68	3.49	25.9
DETP (ng/mL)	Case	259	0.594	41.5	<LOQ	<LOQ	<LOQ	1.37	25.0
	Sub-cohort	548	0.594	47.4	<LOQ	<LOQ	<LOQ	1.58	131
DEDTP (ng/mL)	Case	259	0.594	0.38	<LOQ	<LOQ	<LOQ	<LOQ	<LOQ
	Sub-cohort	548	0.594	0.18	<LOQ	<LOQ	<LOQ	<LOQ	<LOQ
Sum DEP * (nmol/L)	Case	259	NA	NA	2.04	10.1	18.2	34.3	241
	Sub-cohort	547	NA	NA	0.02	11.5	19.7	37.3	581
DMP (ng/mL)	Case	259	3.003	47.7	<LOQ	<LOQ	<LOQ	7.01	91.8
	Sub-cohort	547	3.003	52.3	<LOQ	<LOQ	3.27	10.2	168
DMTP (ng/mL)	Case	259	0.429	89.6	<LOQ	0.91	2.09	5.18	68.6
	Sub-cohort	548	0.429	93.3	<LOQ	1.05	2.58	8.06	221
DMDTP (ng/mL)	Case	259	1.320	13.5	<LOQ	<LOQ	<LOQ	<LOQ	37.6
	Sub-cohort	548	1.320	16.9	<LOQ	<LOQ	<LOQ	<LOQ	83.9
Sum DMP * (nmol/L)	Case	259	NA	NA	3.14	25.6	56.7	114	1363
	Sub-cohort	547	NA	NA	0.05	31.7	66.1	156	1979

Note: DEP, diethylphosphate; DETP, diethylthiophosphate; DEDTP, diethyldithiophosphate; DMP, dimethyphosphate; DMTP, dimethylthiophosphate; DMDTP, dimethyldithiophosphate. * Values for concentrations below the LOQ were imputed. Values standardized to the geometric mean of specific gravity.

**Table 4:** Associations between prenatal urinary organophosphorus-pesticide metabolite concentrations and child’s preschool ADHD in a nested case–cohort study of the Norwegian Mother, Father and Child Cohort (MoBa), birth years 2004–2008.

OP Metabolite	Case	Sub-Cohort	Log_10_OR (95% CI)	Case	Sub-Cohort	Q1 (Ref) OR (95% CI)	Case	Sub- Cohort	Q2 OR (95% CI)	Case	Sub-Cohort	Q3 OR (95% CI)	Case	Sub-Cohort	Q4 OR (95% CI)
∑DEP^0^	259	547	0.76 (0.52, 1.09)	72	129	ref	62	140	0.79 (0.52, 1.20)	67	135	0.89 (0.59, 1.34)	58	143	0.73 (0.48, 1.11)
∑DEP^1^	231	483	1.08 (0.67, 1.75)	66	117	ref	56	124	0.96 (0.59, 1.56)	58	115	1.13 (0.69, 1.85)	51	127	0.94 (0.55, 1.61)
∑DMP^0^	259	547	0.65 (0.49, 0.88)	77	124	ref	63	139	0.73 (0.48, 1.10)	67	135	0.80 (0.53, 1.20)	52	149	0.56 (0.37, 0.86)
∑DMP^1^	231	483	0.79 (0.53, 1.17)	73	111	ref	52	127	0.75 (0.47, 1.19)	59	119	0.88 (0.54, 1.42)	47	126	0.75 (0.44, 1.26)

Note: Log_10_OR, OR per log_10_-unit increase in concentration of OP metabolite; ref, reference; Q1, quartile 1; Q2, quartile 2; Q3, quartile 3; Q4, quartile 4; CI, confidence interval; ∑ DEP, sum of diethylphosphates metabolites; ∑ DMP, sum of dimethylphosphates metabolites; OR, odds ratio. Model 0, unadjusted; Model 1 adjusted for maternal education, parity, maternal income dependency, maternal age, marital status, maternal ADHD-like symptoms, pesticide use, fruit consumption, raw vegetable consumption, and season; all adjusted models were mutually adjusted for complementary DAP metabolite group.

**Table 5:** Assessment of modification by variation in PON1 single-nucleotide polymorphism genotypes and child sex.

Genotypes	*n* Cases/Sub-Cohort	Log_10_DEP (OR 95% CI)	*p*-Interaction	Log_10_DMP (OR 95% CI)	*p*-Interaction
PON1 L55M (maternal rs854560)					
MM (AA)	100/213	0.67 (0.35, 1.27)	0.40	0.66 (0.40, 1.11)	0.77
LM (TA) or LL (TT)	144/306	0.94 (0.58, 1.51)		0.73 (0.49, 1.09)	
PON1 Q192R (maternal rs662)					
QQ (TT)	124/279	0.74 (0.44, 1.26)	0.43	0.68 (0.45, 1.04)	0.72
QR (TC) or RR (CC)	123/243	1.01 (0.57, 1.79)		0.76 (0.48, 1.21)	
Child sex at birth					
Male	144/271	0.73 (0.43, 1.24)	0.45	0.74 (0.54, 1.00)	0.83
Female	114/267	0.98 (0.57, 1.69)		0.72 (0.47, 1.10)	

Note: PON1, paraoxonase 1; DEP, diethylphosphate metabolites; DMP, dimethylphosphate metabolites; OR, odds ratio; p-interaction, *p*-value for the interaction term; each model adjusted for specific gravity, maternal age, maternal education level, parity (reduced to most parsimonious model); models were mutually adjusted for complementary DAP metabolite group.

## Data Availability

Research data used in this paper may be requested directly from the Norwegian Mother, Father, and Child cohort study (mobaadmin@fhi.no) in accordance with the standard MoBa guidelines for data access. For more information on data access procedures, please see www.fhi.no/en/studies/moba/for-forskere-artikler/research-and-data-access/ (accessed on 26 May 2022).

## Supplementary Materials

The following supporting information can be downloaded at: https://www.mdpi.com/article/10.3390/ijerph19138148/s1, Table S1. Prenatal urinary organophosphorus pesticide metabolite concentration (nM/L) distribution in a nested case–cohort study of preschool attention-deficit hyperactivity disorder in the Norwegian Mother and Child Cohort (MoBa), birth years 2004–2008; Table S2. Associations, based on alternative adjustment sets, between prenatal urinary organophosphorus pesticide metabolite concentrations and child’s preschool ADHD in a nested case–cohort study of the Norwegian Mother, Father and Child Cohort (MoBa), birth years 2004–2008.
