# Supplementary material for: Prenatal Exposure to Organophosphorus Pesticides and Preschool ADHD in the Norwegian Mother, Father and Child Cohort Study
Source: Int J Environ Res Public Health. 2022 Jul 2;19(13):8148. doi: 10.3390/ijerph19138148 (PMC9266339; doi:10.3390/ijerph19138148)
Supplement: Supplementary file 1 [file ijerph-19-08148-s001.zip › ijerph-1766881-supplementary.pdf]

Supplementary Material

Table S1. Prenatal urinary organophosphorus pesticide metabolite concentration (nM/L) distribution in a nested case–cohort study of preschool attention-deficit hyperactivity disorder in the Norwegian Mother and Child Cohort (MoBa), birth years 2004–2008.

OP Metabolite	Population	*N*	Geometric Mean	Geometric SD	Min	25%	50%	75%	Max
DEP (nM/L)	Case	259	13.5	2.31	1.15	7.71	12.9	23.4	162
	Subcohort	547	14.5	2.40	0.01	8.52	14.2	24.7	124
DETP (nM/L)	Case	259	3.57	4.07	0.12	1.14	3.61	9.38	184
	Subcohort	547	4.33	4.14	0.01	1.63	4.18	10.5	541
ΣDEP (nM/L)	Case	259	19.0	2.46	2.04	10.1	18.2	34.3	241
	Subcohort	547	21.1	2.58	0.02	11.5	19.7	37.3	581
DMP (nM/L)	Case	259	25.0	3.66	0.98	7.31	28.4	60.6	551
	Subcohort	547	31.6	3.74	0.03	11.0	37.5	80.2	1057
DMTP (nM/L)	Case	259	20.0	3.49	0.27	8.13	19.5	43.0	785
	Subcohort	547	26.5	3.88	0.01	10.4	22.9	60.2	1291
DMDTP (nM/L)	Case	259	2.86	3.34	0.19	1.33	2.41	5.05	276
	Subcohort	547	3.33	3.62	0.01	1.43	2.76	5.93	356
ΣDMP (nM/L)	Case	259	56.1	3.05	3.14	25.6	56.7	114	1363
	Subcohort	547	72.0	3.29	0.05	31.7	66.1	156	1979

Note: DEP, diethylphosphate; DETP, diethylthiophosphate; ΣDEP, sum of diethylphosphates; DMP, dimethyphosphate; DMTP, dimethylthiophosphate; DMDTP, dimethyldithiophosphate; ΣDMP, sum of dimethylphosphates. Values for concentrations below the LOQ were imputed. Values standardized to the geometric mean of specific gravity.

Table S2. Associations, based on alternative adjustment sets, between prenatal urinary organophosphorus pesticide metabolite concentrations and child's preschool ADHD in a nested case–cohort study of the Norwegian Mother, Father and Child Cohort (MoBa), birth years 2004–2008.

OP Metabolite	Case	Sub-Cohort	Log$_{10}$OR (95% CI)	Case	Sub-Cohort	Q1 (ref) OR (95% CI)	Case	Sub-Cohort	Q2 OR (95% CI)	Case	Sub-Cohort	Q3 OR (95% CI)	Case	Sub-Cohort	Q4 OR (95% CI)
∑DEP[0]	259	547	0.76 (0.52 1.09)	72	129	ref	62	140	0.79 (0.52, 1.20)	67	135	0.89 (0.59, 1.34)	58	143	0.73 (0.48, 1.11)
∑DEP[1]	231	483	1.08 (0.67, 1.75)	66	117	ref	56	124	0.96 (0.59, 1.56)	58	115	1.13 (0.69, 1.85)	51	127	0.94 (0.55, 1.61)
∑DEP[2]	258	538	1.07 (0.69, 1.67)	71	129	ref	62	139	0.91 (0.59, 1.42)	67	131	1.13 (0.72, 1.78)	58	139	0.97 (0.60, 1.58)

∑DEP[3]	250	526	1.09 (0.69, 1.72)	69	125	ref	62	137	0.93 (0.59, 1.46)	64	127	1.10 (0.69, 1.74)	55	137	0.94 (0.57, 1.54)
∑DEP[4]	256	526	1.08 (0.69, 1.69)	71	127	ref	62	137	0.81 (0.52, 1.25)	66	127	0.85 (0.54, 1.34)	57	135	0.66 (0.40, 1.08)
∑DEP[5]	244	503	1.10 (0.69, 1.75)	69	120	ref	60	131	0.95 (0.60, 1.52)	61	122	1.10 (0.68, 1.76)	54	130	0.96 (0.58, 1.60)
∑DMP[0]	259	547	0.65 (0.49, 0.88)	77	124	ref	63	139	0.73 (0.48, 1.10)	67	135	0.80 (0.53, 1.20)	52	149	0.56 (0.37, 0.86)
∑DMP[1]	231	483	0.79 (0.53, 1.17)	73	111	ref	52	127	0.75 (0.47, 1.19)	59	119	0.88 (0.54, 1.42)	47	126	0.75 (0.44, 1.26)
∑DMP[2]	258	538	0.72 (0.51, 1.04)	77	123	ref	62	138	0.80 (0.52, 1.23)	67	133	0.87 (0.56, 1.35)	52	144	0.66 (0.41, 1.08)
∑DMP[3]	250	526	0.71 (0.49, 1.02)	76	120	ref	59	133	0.81 (0.52, 1.25)	65	131	0.85 (0.54, 1.34)	50	142	0.66 (0.40, 1.08)
∑DMP[4]	256	526	0.75 (0.52, 1.08)	77	122	ref	61	136	0.92 (0.59, 1.45)	67	129	1.14 (0.72, 1.81)	51	139	0.96 (0.59, 1.57)
∑DMP[5]	244	503	0.77 (0.53, 1.11)	76	116	ref	56	130	0.79 (0.50, 1.23)	63	126	0.87 (0.55, 1.39)	49	131	0.73 (0.44, 1.22)

Note: $Log_{10}OR$, OR per log_{10}-unit increase in concentration; ref, reference; Q1, quartile 1; Q2, quartile 2; Q3, quartile 3; Q4, quartile 4; CI, confidence interval; ∑DEP, sum of diethylphosphates metabolites; ∑DMP, sum of dimethylphosphates metabolites; OR, odds ratio. model 0, unadjusted; model 1 adjusted for maternal education, parity, maternal income dependency, maternal age, marital status, maternal ADHD-like symptoms, pesticide use, fruit consumption, raw vegetable consumption, and season; model 2 adjusted for maternal age, maternal education level, parity; model 3 adjusted for maternal income dependency, maternal education, and parity; model 4 adjusted for maternal age, maternal education, maternal ADHD-like symptoms, and parity; model 5 adjusted for maternal education, parity, maternal income dependency, maternal age, maternal ADHD-like symptoms, fruit consumption, and raw vegetable consumption; all models adjusted for specific gravity through standardization; All adjusted models were mutually adjusted for complementary DAP metabolte group.
